# Health Communication in Times of Pandemics: A Framework for Increased Community Participation in Infection Prevention

**DOI:** 10.3390/ijerph22091398

**Published:** 2025-09-07

**Authors:** Ahmed Alobaydullah, Andrew Scott LaJoie

**Affiliations:** 1 Emergency Medical Services Program, College of Applied Medical Sciences, King Saud bin Abdulaziz University for Health Sciences, Al Ahsa 31982, Saudi Arabia; 2 King Abdullah International Medical Research Center, Al Ahsa 31982, Saudi Arabia; 3 Ministry of the National Guard—Health Affairs, Al Ahsa 31982, Saudi Arabia; 4 Department of Health Promotion and Behavioral Sciences, University of Louisville, Louisville, KY 40202, USA; scott.lajoie@louisville.edu

**Keywords:** preventive behaviors, risk communication, social cognitive theory, non-pharmaceutical interventions, conceptual framework

## Abstract

Introduction: Pandemic communication faces significant challenges due to the dynamic nature of disease outbreaks, societal influences, and evolving communication platforms. Effective non-pharmaceutical interventions (NPIs) depend on robust health communication strategies. This study aims to develop a conceptual model to guide NPIs communication during pandemics, grounded in widely applied risk communication theories. Methods: Using Jabareen’s conceptual framework analysis method, this study synthesized interdisciplinary literature from public health, psychology, and risk communication. The method involves mapping data sources and concept categorization and integration. We examined Crisis and Emergency Risk Communication (CERC), the Social Amplification of Risk Framework (SARF), and Social Cognitive Theory (SCT) to develop a comprehensive NPIs communication framework. Results: The Pandemic Behavioral Prevention Framework delineates pandemic communication into five phases: pre-crisis, initial event, maintenance, resolution, and evaluation. It emphasizes targeting vulnerable populations, addressing trust deficits, and leveraging effective communication channels. Key concepts such as self-efficacy, vicarious learning, and social risk amplification are integrated to enhance public adherence to NPIs. Conclusion: The framework bridges gaps in pandemic communication by integrating risk and health communication principles, fostering trust, and addressing social determinants of health. It highlights the importance of pre-crisis education and the utilization of social media for targeted messaging.

## 1. Introduction

Communicating risk during disasters is always a significant challenge for public health researchers, policymakers, and practitioners. For researchers, understanding how people perceive risk requires analyzing the many factors that shape these perceptions. These factors include resistance to pharmaceutical interventions (e.g., vaccines), the spread of misinformation, cultural influences, health literacy levels, and alternative worldviews [1,2]. Policymakers and organizations face a similar challenge. They must establish clear communication channels, provide accurate and timely recommendations, maintain public trust, navigate evolving media landscapes, and craft effective risk messages [3]. Meanwhile, practitioners must quickly and clearly convey complex information to audiences who are encountering the risk and uncertainty [4].

These challenges highlight the difficulty of building community resilience during pandemics and encouraging preventive behaviors like handwashing and mask-wearing. Since public health recommendations rely on persuasion rather than mandates, communicators must use theory and real-time assessment to guide their messaging [5]. Theories give communicators a framework to work from, helping them figure out what to say and how to say it [6]. At the same time, each pandemic presents unique challenges. During the 2004 Severe Acute Respiratory Syndrome (SARS) outbreak, the demand for information far outpaced its availability [7]. The 2009 H1N1 pandemic saw a surge in rumors, which negatively affected trust in health authorities, as unclear messaging left room for speculation [1]. The rise of social media further complicated risk communication during COVID-19, amplifying misinformation and making it harder to control the narrative [8]. COVID-19 posed new challenges for health communicators, especially in early 2020. A perceived lack of government control fueled public anxiety, leading some to act against their own interests [9]. Objections to guidelines adherence occurred in countries such as the United Kingdom and Canada, the anti-vaccine movement regained momentum, and disparities in adherence to preventive measures persisted [10,11].

Pandemics typically begin with uncertainty, limited evidence on intervention effectiveness, and the absence of a vaccine or treatment [12]. In these early stages, health communicators must work with incomplete data to promote non-pharmaceutical interventions (NPIs) and curb disease spread. Effective communication is crucial, and a conceptual model can provide public health professionals with a structured approach to risk messaging at the onset of a pandemic. This paper provides a conceptual model to explain the decision-making processes behind recommending non-pharmaceutical interventions (NPIs) during pandemics. By critically evaluating key risk communication theories and incorporating lessons from COVID-19, this model provides a structured framework for public health professionals to enhance messaging strategies in future health crises.

## 2. Methods

To explore how risk is communicated during pandemics, this study draws on Jabareen’s conceptual framework analysis, which is rooted in grounded theory [13]. This approach is designed to identify, develop, and connect key concepts that collectively form a theoretical framework for understanding a phenomenon. Unlike a mere collection of related concepts, a conceptual framework is “a network, or a plane, of interlinked concepts that together provide a comprehensive understanding of a phenomenon or phenomena [13]”. Because the communication landscape shifts during a pandemic, this flexible approach is well-suited for analyzing how messaging evolves over time.

**Figure 1:**
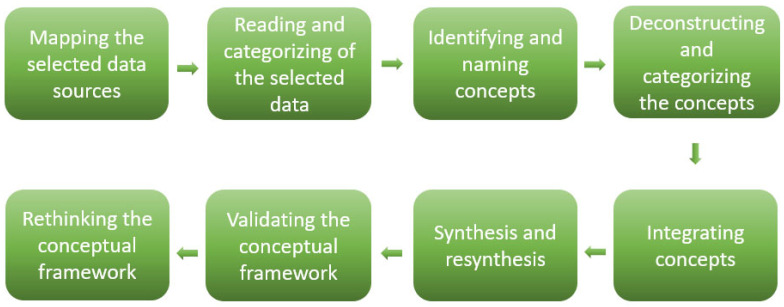
Jabareen’s conceptual framework analysis methodology.

Jabareen’s conceptual framework analysis consists of eight phases: (1) mapping data sources from multiple disciplines, (2) conducting a literature review and categorizing data, (3) identifying and labeling relevant concepts, (4) deconstructing and organizing these concepts, (5) integrating them into a coherent structure, (6) synthesizing and ensuring consistency, (7) validating the framework, and (8) refining the framework as needed [12]. To better align with the current study’s objectives, some of these phases were condensed during analysis [Figure 1].

Each concept within the framework is examined in terms of its distinct characteristics, assumptions, limitations, and role in shaping the broader understanding of risk communication. The framework development process involves a comprehensive review and categorization of literature spanning the psychological, environmental, and social dimensions of risk communication. To build a strong multidisciplinary base, the framework incorporates literature from fields like public health, psychology, and communication studies.

### 2.1. Phases 1 and 2: Mapping and Categorizing Selected Data Sources—Theoretical Background

The first stages of this study involved reviewing and categorizing multidisciplinary literature to construct a conceptual framework for pandemic risk communication [14]. This framework is grounded in three key theoretical approaches that inform the communication of non-pharmaceutical interventions (NPIs) during pandemics. The first approach is the Crisis and Emergency Risk Communication (CERC) model, developed by the CDC, which provides a structured communication strategy applicable to all phases of a pandemic. The second approach is the Social Amplification of Risk Perception Framework (SARF), which highlights how media strategies shape public risk perception and influence communication effectiveness. The third approach is Social Cognitive Theory (SCT), which examines the reciprocal relationship between individuals and their environment, emphasizing community participation as a fundamental motivator for adopting preventive behaviors. Together, these theories build a more complete picture of how pandemic risk is communicated, each adding different insights on behavior, messaging, and perception.

#### 2.1.1. Phases of Crisis and Emergency Risk Communication

Promoting time-sensitive behaviors such as NPIs requires a well-structured communication strategy that is responsive to the different phases of a pandemic [15]. CERC stands out from other crisis models because it is more adaptable and accounts for the many layers of a public health emergency. Unlike the Three-Stage Model of Crisis Communication and Turner’s Six-Stage Man-Made Disasters model, which are often criticized for being overly simplistic or lacking specificity, the CERC model offers a more adaptable structure that considers the evolving nature of pandemics [16,17]. The CERC model’s strength lies in its ability to account for multiple dimensions of a pandemic, including its physical, health, social, and psychological impacts [15].

A common pitfall in pandemic preparedness is the assumption that future risk can be accurately anticipated based on past events. This assumption often leads to inadequate responses when confronted with unprecedented crises [18]. Preparing communities for NPIs requires proactive engagement across all phases of a pandemic to enhance long-term resilience [19]. The CERC model is based on two fundamental assumptions. First, it conceptualizes pandemics as complex, dynamic events influenced by multiple interacting factors rather than simple cause–effect relationships [20]. Second, it acknowledges that pandemics are time-order and time-sensitive events shaped by how individuals experience and recall them [21]. Personal narratives of the COVID-19 pandemic illustrate this phenomenon, with affected individuals often describing their experiences in ways that reflect deep psychological and social disruptions. One participant, for instance, recalled that “it felt like time stood still for many months while life was ‘on hold’ and every day was similar, with no punctuation by landmark events [22] (p 1137)”. This time-oriented perception of pandemics underscores the necessity of adaptive communication strategies that resonate with the lived experiences of the public.

#### 2.1.2. Socially Amplified Pandemic Risk Perception

The Social Amplification of Risk Framework (SARF) explains how risk perception is shaped by both social and individual processes [23]. Since its initial conceptualization,^24^ SARF has been widely applied to assess how public and individual responses to risk are influenced by various amplification channels, including social organizations, media platforms, and interpersonal interactions. The framework draws on classical communication theory, where amplification refers to the intensification or attenuation of a transmitted message, altering the amount of information retained from the original source [24]. SARF posits that risk messages interact with psychological, social, and cultural factors, ultimately shaping behavior through a collectively constructed perception of risk [23]. From this perspective, experiencing risk extends beyond the immediate physical threat of infection; it is also influenced by the subjective meanings and interpretations ascribed by individuals and communities [25].

Risk communicators must recognize that the public’s perception of risk is not merely an inferior version of the expert’s objective assessment. Rather, SARF provides communicators with opportunities for intervention by identifying key points where risk perceptions can be influenced [26]. The amplification or attenuation of risk occurs in two stages: the initial dissemination of information by official or unofficial sources, followed by the societal response, in which individuals translate risk messages into behavioral, economic, and social actions [27]. Early messaging is affected by how much information is shared, how consistent and clear it is, and whether it feels exaggerated or emotionally charged [25]. As individuals receive and process NPIs-related information, they become amplification stations, further shaping public perception through their behavioral responses and interpersonal communication [28]. These amplification stations, whether individuals, groups, or institutions, play a critical role in constructing collective risk perceptions.

SARF also highlights the conditions under which social amplification is most pronounced. Risk perception tends to be amplified or heightened in situations characterized by high uncertainty and perceived personal threat, where individuals rely more on external information sources than on prior knowledge [23,29]. In the context of NPIs communication, SARF informs the understanding of how individuals cognitively and affectively process messages and develop stable beliefs regarding preventive behaviors. This framework accounts for the transformation of individual perceptions into shared social understandings of risk [28].

A crucial component of SARF is its attention to how small groups such as marginalized and at-risk populations have a different risk perception than the general population. The inclusion of message targeting ensures that risk messages resonate with specific population segments based on shared characteristics such as race, socioeconomic status, and health vulnerabilities [30]. Before implementing targeted messages, communicators must conduct audience segmentation to identify the most vulnerable groups and tailor messages to their needs and concerns [31]. The COVID-19 pandemic demonstrated the importance of this approach, as African American populations in the United States experienced disproportionately higher fatality rates compared to White populations [32]. This disparity cannot be attributed solely to race or access to healthcare but must also consider underlying health conditions such as hypertension, diabetes, and cardiovascular disease [32,33]. Effective NPIs communication requires a deliberate focus on these at-risk groups, ensuring that they receive comprehensive and contextually relevant information to guide their behavioral decisions.

At the same time, communicators need to be alert to the potential stigmatizing effects of targeted messaging. The 2009 H1N1 pandemic had the unfortunate moniker “swine flu”; U.S.-based agricultural labor, especially Mexican migrant or seasonal farmworkers, experienced significant discrimination from people who mistakenly attributed the cause of the pandemic to pork processing [34]. Asians around the world experienced discrimination and violence during the COVID-19 pandemic due to its origination in China [35,36].

#### 2.1.3. Social Cognitive Influences on NPIs Behavior

**Figure 2:**
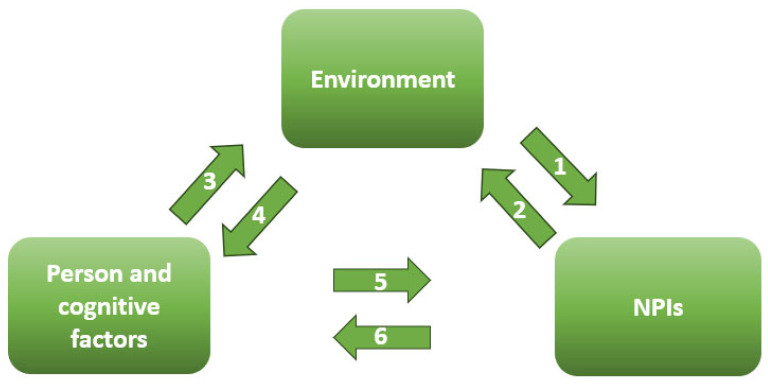
Bandura’s triad of reciprocal determinism.

Social Cognitive Theory (SCT) plays a critical role in this framework by explaining the triadic reciprocal determinism that shapes NPIs adoption. This dynamic process involves the interaction of behavior, environmental influences, and individual psychological processes [37]. While CERC and SARF address the timing and cognitive mechanisms of risk communication, SCT provides insight into how personal and environmental factors interact to influence behavior [Figure 2].

The interactions between Bandura’s triad show up in many forms, as behavior, thoughts, and environment keep influencing each other. The social, economic, and political context can prompt individuals to adopt NPIs, while personal behaviors, such as aligning with a political ideology, can shape the broader environment. Individuals also influence their sociocultural surroundings through shared beliefs and collective cognition. At the same time, environmental factors can alter cognitive attributes like self-efficacy, just as a person’s cognitive state governs their engagement with preventive behaviors. In turn, sustained adherence to NPIs can further influence one’s cognitive perceptions, creating a continuous feedback loop [38]. Risk communication strategies primarily target personal and cognitive factors, aiming to drive changes in the other components of this triadic system [39]. This framework positions the individual as the central focus of NPIs messaging. By improving knowledge, enhancing self-efficacy, and fostering positive outcome expectancies, pre-pandemic education and real-time messaging can strengthen public engagement with NPIs. Since self-efficacy is enhanced through vicarious learning and verbal persuasion, communicators can use role models and authoritative figures to reinforce NPIs adoption [40,41].

### 2.2. Phase 3: Identifying and Naming Concepts

In this phase, the theoretical models previously discussed were examined in greater depth through an extensive review of relevant literature. Once their relevance to pandemic mitigation was established, the next step involved identifying specific concepts that influence health communication during pandemics. Using CERC as a base, we adopted its phase structure to match how messaging around NPIs changes throughout a pandemic [42] [Table 1].

To effectively promote NPIs, health communicators must understand not only how individuals receive and interpret messages but also how they act as amplification or attenuating points within their social networks. The Social Amplification of Risk Framework (SARF) serves here as a conceptual lens through which these amplification processes are understood. We adopt and adapt the steps outlined by Renn et al. [28] to describe how individuals process information and how these insights can be applied to the targeted communication of NPIs among specific subgroups [Table 2].

### 2.3. Phases 4 and 5: Deconstructing, Categorizing, and Integrating the Concepts

Building on the concepts identified in the previous phase, this step involved organizing and integrating them into a unified framework. Each concept was categorized according to its ontological, epistemological, or methodological role within the overall structure [13]. Certain concepts, such as self-efficacy, were found to be central across all three theoretical models. These shared concepts were defined and positioned within the conceptual framework according to their distinct functions and theoretical contributions [Table 3].

### 2.4. Phase 6: Synthesizing, Re-Synthesizing, and Ensuring Coherence

This stage involved synthesizing the conceptual elements into a coherent whole. To ensure the framework coherence, the different concepts were adjusted and aligned more tightly. The final framework segments NPIs communication into five phases, each marked by specific messaging strategies and expected audience behaviors. The framework centers on vulnerable populations, positioning them as a core focus of pandemic communication efforts and emphasizing their role in determining the success of public health interventions.

### 2.5. Phases 7 and 8: Validating and Rethinking the Conceptual Framework

In the final phases, the PBPF undergoes preliminary validation through evaluation by public health practitioners unaffiliated with its development. The goal of these evaluations is to see if the framework holds together, covers the key areas, and makes sense in real-world settings. As part of this process, we plan to present the framework to larger audiences, including at academic conferences and scientific meetings. Open-ended feedback will be solicited regarding its feasibility, utility, and areas for refinement. This input will guide future iterations of the framework and support its continued development.

## 3. Results

**Figure 3:**
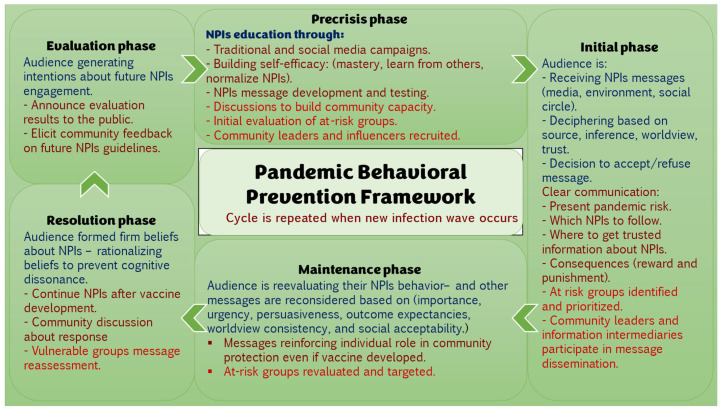
Pandemic Behavioral Prevention Framework.

The ***Pandemic Behavioral Prevention Framework*** is designed to address all layers of an infectious disease crisis by structuring the response into five distinct phases, shaping appropriate and socially accepted risk perceptions, and prioritizing the most vulnerable populations. An integral part of the success of this framework is the recruitment of credible, trustworthy, communicators; these are the leaders of the community, the local public health agency team, and local gatekeepers. Locality here is important; the local public health agency is often the most informed entity of the community needs, and their strong ties to the public should be in-place [Figure 3].

In a future pandemic, public health teams could start by using familiar voices and social media to help people understand and accept protective behaviors like mask-wearing and handwashing. As the situation unfolds, clear and trustworthy messages would guide people on what to do, why it matters, and where to obtain reliable information. Over time, as fatigue sets in, reminders would help people stay motivated, especially those at higher risk. When vaccines or treatments become available, the focus would shift to addressing concerns and encouraging informed decisions. Finally, listening to the public’s experiences and feedback would help improve future responses. This step-by-step approach keeps communication grounded, responsive, and people-centered through every phase of the crisis.

## 4. Discussion—Lessons Learned

The ***Pandemic Behavioral Prevention Framework*** is designed with an awareness of how audiences process information, ensuring that communication efforts align with individuals’ cognitive, affective, and behavioral responses at different pandemic phases. For example, during the maintenance phase (e.g., past the peak of the epidemic curve), health communication is strategically directed to reinforce continued adherence to NPIs. Additionally, the framework prioritizes identifying and targeting vulnerable populations as a fundamental component of effective pandemic communication. The proposed framework tries to fill in the gaps that past pandemic messaging efforts often missed.

### 4.1. Integrating Risk and Emergency Communication with Health Communication

During infectious disease outbreaks, public health organizations rely primarily on two established frameworks: the Crisis and Emergency Risk Communication (CERC) model developed by the CDC and the Outbreak Communication Guidelines established by the WHO [44]. These models were designed to manage the urgency, uncertainty, and time constraints that define health crises [15]. Their relevance is evident when considering the crises that shaped their development, such as the 2001 World Trade Center attacks and the anthrax bioterrorism incidents [45]. However, pandemics pose distinct challenges that go beyond isolated bioterrorism events, as they are ongoing, unpredictable, and spread rapidly across global populations [45].

Existing risk and emergency communication frameworks are well-suited for the early stages of an outbreak, when containment efforts focus on minimizing harm at the community level. However, these models are less effective in managing the prolonged and unpredictable nature of pandemics [46]. The COVID-19 pandemic highlighted the limitations of traditional crisis communication approaches, as the virus continuously mutated, altered transmission patterns, and created varying degrees of disease severity across different regions and timeframes [47]. To close that gap, the PBPF brings in communication strategies that begin even before a crisis starts, aiming to build up public resilience ahead of time. Establishing behavioral norms such as proper cough etiquette, hand hygiene, and acceptance of NPIs before a crisis emerges can enhance public responsiveness and preparedness during a pandemic [46]. Post-pandemic communications should highlight evidence that reinforces the effectiveness of the promoted behavior.

### 4.2. Utilizing Effective Communication Channels

As pandemics spread rapidly, communication efforts must leverage fast and adaptive channels. The 2009 H1N1 pandemic was the first to unfold with widespread global access to the Internet, revealing both the potential and challenges of online communication [48]. However, public health communicators struggled to adapt to the Internet’s disruption of traditional sender–receiver communication models. The emergence of blogs, social media, and digital platforms removed the clear line between expert-driven communication and user-generated content, challenging the authority of public health messaging [49]. The COVID-19 pandemic further exacerbated these challenges, as social media became the dominant space where individuals received, transmitted, and discussed pandemic-related information [50].

Effective pandemic communication must take place on platforms where the public is already engaging. Due to the widespread connectivity, an infodemic took off and often spread faster than official health advice could keep up [50]. Additionally, the blurred boundaries between health professionals’ personal and professional identities have created a disconnect between expert opinion and public perception [51]. The ***Pandemic Behavioral Prevention Framework (PBPF)*** emphasizes the need to fully integrate widely used digital communication platforms into risk messaging strategies to enhance reach and effectiveness.

### 4.3. Building Trust Before It Is Compromised

Building trust is a core objective of risk communication. Both the CDC and WHO recognize it as the foundation of effective public health messaging [15,44]. Trust is often regarded as the most valuable asset in a crisis; once lost, efforts to persuade the public to adopt preventive behaviors become significantly more difficult [52]. However, trust cannot be built spontaneously during an emergency; it is largely shaped by society’s pre-existing confidence in institutions and leadership [53].

In modern societies, public trust in policymakers and governmental institutions has been steadily declining, leading to what some describe as post-trust societies [53,54]. A failure to establish trust before a crisis undermines public confidence in official risk communication efforts, as seen during COVID-19, where inconsistent messaging and political divisions heightened public skepticism [55]. Trust is built through objectivity, openness, honesty, competency, fairness, and consistency, all of which must be demonstrated in long-term public health communication strategies [56]. The ***Pandemic Behavioral Prevention Framework*** supports pre- and post-crisis trust-building through education and calls for transparent, clear messaging throughout all pandemic phases. Political environments further complicate trust-building efforts. How well a country manages a pandemic often depends on its political stability, leadership systems, and how its policies are put into action [57]. While this study focuses on individual behaviors rather than policy reforms, it acknowledges the political dynamics that shape public engagement with NPIs.

### 4.4. Limiting the Consequences of Poor Responses for Vulnerable Populations

As with previous pandemics, COVID-19 disproportionately affected the most vulnerable populations [58]. The pandemic exacerbated existing social, economic, and health inequities, placing marginalized communities at greater risk of severe illness and mortality [58]. These disparities transcended national borders, affecting both high-income and low-income countries, highlighting the universal nature of pandemic-related vulnerabilities [59].

The burden of pandemic response should not fall solely on at-risk individuals. Unlike other health crises that require access to specialized medical care or financial resources, NPIs involve behavioral changes with minimal material barriers. Addressing social determinants of health (SDOH) in pandemic response efforts is critical for ensuring equitable health outcomes [60]. Effective communication is a key part of pandemic response, as it helps achieve satisfactory levels of testing, screening, and following safety guidelines [60]. Health systems traditionally focus on structural factors such as healthcare access, but risk communication must be prioritized to prevent increasing health disparities [61]. The ***Pandemic Behavioral Prevention Framework*** underscores the importance of targeting underserved populations with tailored risk messages to mitigate the disproportionate impact of pandemics.

## 5. Strengths and Limitations

This study introduces a flexible grounded theory approach to develop a conceptual framework for NPIs communication in complex pandemic settings. A key strength of the PBPF is its focus on tailored messaging for vulnerable populations. While not yet tested in practice, this framework creates opportunities for future application, validation, and refinement through empirical studies and expert feedback, enhancing its relevance and impact in real-world public health contexts.

## 6. Conclusions

This paper integrates insights from multiple disciplines to enhance understanding of how to motivate individuals to adopt and maintain NPIs during a pandemic. The ***Pandemic Behavioral Prevention Framework*** provides a structured approach that incorporates how individuals process risk communication and interact with their environment across all pandemic phases. By equipping health communicators with a flexible and applicable tool, this framework offers a practical method for addressing the unpredictable nature of pandemics. Future research should test the framework in real-world applications through experimental studies to assess its impact on public adherence to NPIs.

## Figures and Tables

**Table 1:** NPIs pandemic communication according to CERC phases.

Phases	Description
Pre-crisis (Risk Messages; Warnings; Preparations)	NPIs communication and education campaigns motivating the public and the response community to Understand pandemic risk and how prevention works. Build capacity to enable behavior change. Test strategies and message content.
Initial Event (Uncertainty Reduction; Self-efficacy; Reassurance)	Rapid communication to the general public to Provide factual information about the pandemic risk to prevent emotional turmoil. Provide specific instructions on which NPIs to follow. Provide continuous communication on where to obtain more information.
Maintenance (Ongoing Uncertainty Reduction; Self-efficacy; Reassurance)	Communication to the general public and to affected groups seeking to facilitate In-depth understanding of the pandemic risk and effectiveness of NPIs reassessed. Reinforcement of the individual role in preventing a pandemic; continuing to build self-efficacy.
Resolution (Updates Regarding Resolution; Discussions about Cause and New Risks/New Understandings of Risk)	Public communication and campaigns directed toward the general public and affected groups seeking to Persuade the public to continue NPIs after vaccine/treatment is disseminated. Engage in community-wide discussions on the overall response. Communicate how NPIs work with new risks.
Evaluation (Discussions of Adequacy of Response; Consensus about Lessons and New Understandings of Risks)	Communication directed toward the community to Evaluate community response. Discuss lessons learned. Improve future responses by evaluating current community actions—link to phase I.

**Table 2:** NPIs pandemic communication according to SARF individual steps to risk perception.

Steps	Description
Passing through attention filters	NPIs messages from the environment, other individuals, and the media are selected and processed.
Decoding of signals	Attention is given to what the messages mean. Message deciphering is dependent on sources of information, explicit or implicit inferences, what factual statements mean to the receiver, and beliefs on the credibility and trust level of the source. In this step, the political environment is considered by the receiver.
Drawing inferences	Conclusions are made about the message and the source; the receiver uses intuitive heuristics to generalize NPIs messages and learned symbolism to judge the significance of the content.
Comparing the decoded messages with other messages	NPIs message is compared with what peers believe about the message, the misinformation in different media outlets, and previous experiences.
Evaluating messages	The messages are weighted based on importance, urgency, persuasiveness, outcome expectancies, perceived consistency with one’s worldview, and social acceptability.
Forming specific beliefs	Firm beliefs about NPIs practice are formed; this is where previously held beliefs are changed or asserted.
Rationalizing belief system	Sorting and reinterpreting NPIs beliefs in order to minimize cognitive dissonance.
Forming a propensity to take corresponding actions	Intentions about future NPIs practices aligned with the belief system are generated.

**Table 3:** Conceptual model integrated concepts.

Concept Name	Definition	Ontology, Epistemology, or Methodology Role	Reference
Pandemic response phases	Response to a pandemic occurs in five phases that cover pre-, during, and post-pandemic efforts.	The phases play a regulatory role in the pandemic efforts, dissecting the response into recognizable chunks.	(M. W. Seeger et al., 2020) [15]
Amplified risk	Risk messages interact with psychological, social, and cultural processes, consequently shaping risk behavior through a socially shaped risk perception.	Inputs from the environment continuously shape risk perception; the nature of risk perceived by the individual is subjective and socially constructed.	(Kasperson et al., 2022) [26]
Self-efficacy	The psychological state concerning one’s perspective on their capacity to execute their plans or successfully accomplish a task.	Belief in one’s ability is found to be a primary motivator for behavior change.	(Bandura, 2004) [37]
Vicarious learning	Observing people in similar situations and believing that the individual can adopt the behavior they observed.	For a behavior to be socially adopted, a vicarious experience must happen, building a sense of self-efficacy and learned mastery.	(Schunk, 2012) [40]
Outcome expectations	The beliefs associated with a specific behavior that result in particular outcomes.	The outcome expected from an action shapes the decision to engage in behavior change. Social influences also shape one’s outcome expectations.	(Zlatanović, 2016) [43]

## Data Availability

No new data were created or analyzed in this study. Data sharing is not applicable to this article.
